# Lower limb deformity due to failed trauma treatment corrected with the Ilizarov technique: Factors affecting the complication rate in 52 patients

**DOI:** 10.3109/17453670903153535

**Published:** 2009-08-01

**Authors:** Hubert J Oostenbroek, Ronald Brand, Peter M van Roermund

**Affiliations:** ^1^Department of Orthopedics, University Medical CentreUtrechtthe Netherlands; ^2^Department of Orthopedics, Leiden University Medical CentreLeidenthe Netherlands; ^3^Department of Medical Statistics, Leiden University Medical CentreLeidenthe Netherlands

## Abstract

**Background and purpose** Failed treatment of fractures may be corrected by the Ilizarov technique but complications are common. In 52 patients with compromised healing of femoral and tibial fractures, the results of secondary reconstruction with Ilizarov treatment were investigated retrospectively in order to identify the factors that contribute to the risk of complications.

**Methods** 52 consecutive patients was analyzed. The median interval between injury and secondary reconstruction was 3 (0.1–27) years. The patients had failed fracture treatment resulting in bone defects, pseudarthrosis, infection, limb length discrepancy (LLD) caused by bone consolidation after bone loss, malunion, soft-tissue loss, and stiff joints. Most patients had a combination of these deformities. The results were analyzed by using logistic regression in a polytomous universal mode (PLUM) logistic regression model.

**Results** The median treatment time was 9 (4–30) months, and the obstacle and complication rate was 105% per corrected bone segment. In 2 patients treatment failed, which resulted in amputation. In all other patients healing of nonunion could be established, malunion could be corrected, and infections were successfully treated. The statistical analysis revealed that relative bone loss of the affected bone was the only predictor for occurrence of complications. From these data, we constructed a simple graph that shows the relationship between relative bone loss of the affected bone and risk of complications.

**Interpretation** Relative bone loss of the affected bone segment is the main predictor of complications after Ilizarov treatment of previously failed fracture treatment. The visualization of the analysis in a simple graph may assist comparison of the complication rates in the literature.

## Introduction

Treatment of the traumatized lower limb rarely fails (Blachut et al. 1997, Bhandari et al. 2000, 2001, Canadian Orthopaedic Trauma Society 2003). High failure rates are only seen in the most severe types of injury, such as open fractures (Gustilo type 3B and 3C). This type of injury frequently ends up with severe deformities (Gopal et al. 2000, Keating et al. 2000). The Ilizarov method may be helpful in treating these post-traumatic deformities including malalignment, nonunion, bone defects, limb length inequality, and osteomyelitis.

The indication for Ilizarov treatment remains limited to the more severe types of injury, as the complication rate during treatment is high and the learning curve is notoriously long (Dahl et al. 1994). For the latter reason, it is not easy for surgeons to gain adequate experience using the Ilizarov method when treating such post-traumatic pathology.

Choosing the Ilizarov method has severe consequences for patients. Disturbance of daily life by the bulky frame, muscle impalement by transfixing wires with associated loss of joint motion, pin-track infections and pain, may all cause great discomfort and psychosocial problems (Ramaker et al. 2000).

The decision to treat with the Ilizarov method must therefore be made after a careful analysis of existing deformities (Paley et al. 1994, Dahl 2000) and sound patient selection with realistic treatment goals; sometimes it is necessary to accept residual shortening, a stiff joint, or other deformity.

We conducted a retrospective study to evaluate the outcome of Ilizarov reconstruction treatment of post-traumatic lower limb deformities in our clinic. In order to understand the outcome achieved, we investigated the statistical relationship between several variables concerning the deformities and the complication rate.

## Patients and methods

### Patients

A retrospective study was performed on 52 patients (33 men) with malunion and nonunion after lower limb trauma. The median age at the start of the Ilizarov treatment was 27 (6–82) years. The median interval between injury and secondary reconstruction was 3 (0.1–27) years (Table 1).

In these 52 patients, 54 corrective procedures in 56 deformed bone segments were done. This means that in one patient (patient 1) the deformity was corrected in 2 sequential procedures, in one patient (patient 42) 2 limbs were corrected at the same time, and in 2 patients (14 and 27) femur and tibia were corrected in the same procedure.

All patients but one (patient 21) had initially been treated in other hospitals. 26 procedures were planned for malunited fractures and 28 for nonunited fractures. Shortening, angulation, and rotational deformity were considered as malunions. Nonunions were deformities with bone defects, or fractures with bone contact that did not unite. 19 nonunions were infected. A median shortening of 4 (1.5–8) cm or 9% (4–21) shortening of the affected bone was found in 25 patients and a bone defect of 8.5 (2–15) cm or 18% (4–29) of the affected bone was found in 18 patients.

### Methods

Deformities were classified according to Dahl et al. (1994). The Ilizarov procedure was aimed at restoring bone continuity, mechanical axis, limb length, and—if applicable—at healing osteomyelitis. When complete correction was not envisaged, a calculated residual deformity was accepted. In these patients, the residual deformity was not calculated as a complication. This strategy was implemented for 5 patients. In patient 1, a two-stage treatment was planned because of a bone defect of 13 cm. Patient 19 had an intraarticular distal femoral fracture with a bone defect of 3 cm. Pre-existing damage to the peri- and intraarticular tissues resulted in a functional knee arthrodesis, which was judged not to improve by bone union. Since a knee arthrodesis requires a limb shortening of 1.5–2 cm, limb lengthening makes no sense. Patient 31 had a 13-cm bone defect that had existed for 15 years. As he was suffering from AIDS, no attempt was made to correct the shortening. The treatment goal in this patient was restoration of bone continuity, permitting him to walk with the help of an orthosis. Patients 29 and 39 had a severe alcohol abuse problem. Thus, the decision was made to treat the bone deformity only and to accept the limb shortening. All patients were treated with a classical circular frame with Ilizarov rings. Bone fixation was performed by using half-pins and tensioned transfixation Kirschner wires. The bone was divided by a multiple-drill hole subperiosteal corticotomy. All lengthenings and corrections were monofocal.

Nonunions and bone defects were treated with trimming and compression of bone ends (Schwartsman et al. 1990, Tucker et al. 1990). In some patients, additional surgical procedures were planned during or after application of the Ilizarov frame.

In treating osteomyelitis, antibiotics were chosen according to culture sensitivity. The duration of the antibiotic treatment was at least 6 weeks.

After removal of the Ilizarov frame, the patients were referred to physiotherapy for further rehabilitation and they were followed up until a stable end-situation was reached. Joint mobility was evaluated at the normal follow-up visits, but not muscle strength.

### Evaluation

Complications of surgical correction were classified according to Paley (1990). We added an extra category to this classification: psychosocial dysfunction, including intoxication states and non-compliant behavior to treatment (Ramaker et al. 2000). This complication may increase the risk of other complications and may change or may lead to premature cessation of the treatment.

### Statistics

Major determinants of the deformities such as large relative bone loss of the affected bone, either shortening or a bone defect, malunion, or (infected) nonunion would be expected to affect the treatment outcome negatively. Other co-existing deformities could influence the outcome as well, and were grouped in a classification as described by Dahl et al. (1994). To assess the relationship between these factors and the complication rate, a polytomous universal mode (PLUM) logistic regression approach was used. Instead of modeling the probability of “complication” as a dichotomy, this approach models the probability of more than 2 ordered outcome categories simultaneously. The independent variables were all added to the model, and then in a backwards stepwise way the multivariate insignificant predictors were removed (relative bone loss, malunion, nonunion, and severity of deformity by the Dahl classification (Dahl et al. 1994)).

The outcome variable was the count of the number of complications and the model estimated the probability of observing 0, 1, 2, or 3 complications as a function of the independent predictors.

## Results

The median external fixator time was 9 (3–30) months. The median amount of lengthening was 3.8 (0.5–15) cm. The median healing index was 2.3 (0.9–9.0) months per cm of lengthening (Table 2).

The defined treatment goal was achieved in 50 patients. Patient 11 could not tolerate the long duration of the treatment. An above-the-knee amputation was done. Due to severe pulmonary disease, further treatment could not be tolerated in patient 49 and a below-the-knee amputation was performed.

14 patients had pin tract infections; these are not considered to be complications by Paley et al. (1990). The treatment led to 27 obstacles and 32 real complications, giving a total complication rate of 105% per treated bone segment.

### Probability of complications

In our study, the minimum number of complications in one patient was 0 and the maximum was 3. The relative amount of bone loss of the affected bone was 4–29%. The statistical analysis showed that relative bone loss, i.e. percentage bone loss in the affected bone, was the single most important statistical determinant of complications. The difference in the coefficient of the main predictor, “bone loss”, between the polytomous analysis and the univariate analysis was small: the coefficient increased from 0.083 to 0.091 by introducing the residual confounding due to not taking into account the other parameters. Thus, the simpler univariate model was chosen to show the magnitude where, given a specific percentage of bone loss, the probabilities of 0, 1, 2, or 3 complications were estimated as an ordered outcome variable. The outcome of this model is visualized as a graph (Figure). It shows the probability of encountering a specific number of complications in relation to the relative amount of bone loss. The standard errors of the estimates that play a role in generating the graph are shown in Table 3. The usual underlying confidence intervals are in Table 3 but not in the graph because the focus of the graph is not on regression lines, but on boundaries (thresholds) between the occurrences of a specific number of complications.

**Figure:**
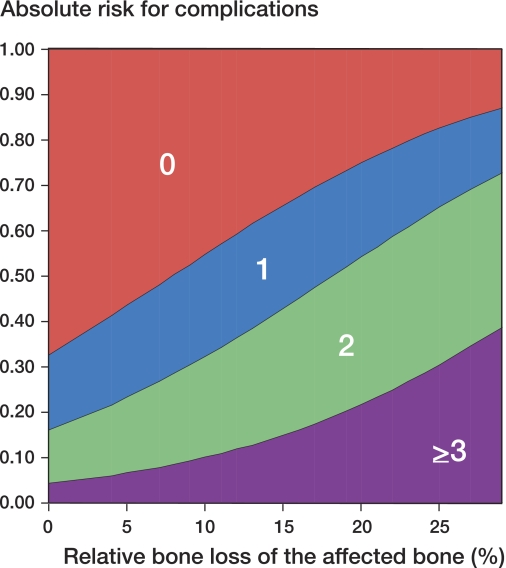
Population estimates of the relation between the percentage bone loss and number of complications in a univariate logistic regression model. The standard errors of the estimates that play a role in the graph are represented in Table 3. The y-axis represents the estimated risk for the amount of complications at a certain relative loss of the affected bone (%) as represented on the x-axis. For example, the risk of having 1 or more complications is 50% for a loss of the affected bone of 8%.

**Table 3.** Population estimates of the relation between relative loss of the affected bone and the number of complications, based on a univariate model. This shows the standard errors of the estimates that play a role in generating the accompanying graph (see figure).

	Estimate	SE	p-value	95% CI
Threshold				
no. of complications = 0	0.712	0.474		-0.218–1.641
no. of complications = 1	1.648	0.518		0.632–2.663
no. of complications = 2	3.099	0.650		1.825–4.374
Location				
relative bone loss	0.091	0.036	0.01	0.021–0.161

The easiest way to interpret the graph is as follows. Imagine a vertical line at a specific percentage of bone loss, say 8%. That line cuts through the boundary between the area of 0 and 1 complications, at precisely 50%. This implies that 50% of the patients with a bone loss of 8% will experience 0 complications (and hence 50% will experience 1 or more complications). Likewise, a vertical line at 18% bone loss will cut the boundary between the area marked as [1] and [2] at 50%; hence, with such a bone loss, 50% of the patients will have at most 1 complication and 50% will have 2 or more complications. At the very end of the graph, the bone loss is assumed to be 29%, the maximum observed in our study. At this point, the probability of experiencing 3 or more complications is 34%, hence 66% of the patients with such a bone loss will have up to 2 complications.

The graph can also be read off horizontally: a horizontal line at 50% complication risk cuts the boundaries at 8% (the 95% CI for this estimate is 1–18%) and 18% (95% CI: 7–29).

## Discussion

In this study of secondary reconstruction for post-traumatic deformities, the treatment goal could be achieved in 50 out of 52 patients. In 5 of these 50 patients, the limb length inequality was intentionally undercorrected—because of patient factors. These patients had problems that should be addressed by limb reconstruction, but at the same time they had problems that would be a contraindication for limb reconstruction, such as AIDS, severe alcohol abuse, and severe behavioral or psychiatric disorders.

Sometimes combined soft tissue, bone, and joint problems cannot all be solved by limb reconstruction or any other treatment. The main goal for these patients should be a stable leg to stand on, without pain, and fit for the application of an orthosis to compensate for the residual deformity.

Adapted treatment goals indicated by concomitant deformities or conditions have rarely been reported (Morandi et al. 1989, Garcia-Cimbrelo and Marti-Gonzalez 2004). There must be an under-reporting, because it is unlikely that this type of patient is not represented in other reconstruction centers. It is undesirable not to report on these patients, because the limitations of treatment or realistic treatment goals of limb reconstruction must be made clear in order not to harm the patient unnecessarily.

In our study, soft tissue problems such as joint contracture or muscle loss could not or could only be partially solved, which corresponds to other reports in the literature (Paley et al. 1990, Dendrinos et al. 1995, Ali and Saleh 2002, Garcia-Cimbrelo and Marti-Gonzalez 2004, Giannikas et al. 2005). Swiontkowski et al. (2002) concluded that soft tissue conditions were prime determinants for reconstruction or amputation of severely traumatized limbs, so it is difficult to understand why the condition of soft tissue as a limitation for treatment outcome is hardly reported.

The treatment resulted in 59 “obstacles” and “real complications”, which represented complication rate of 105% for 56 treated bone segments. This rate is similar to the results reported for limb reconstruction after failed trauma treatment (ranging from 57% to 232%) (Paley et al. 1990, Tucker et al. 1990, Green et al. 1992, Cattaneo et al. 1992, Dendrinos et al. 1995, Marsh et al. 1997, Song et al. 1998, Maini et al. 2000, Paley and Maar 2000, Garcia-Cimbrelo and Marti-Gonzalez 2004, Mahaluxmivala et al. 2005). However, the variation in patient groups is large, making comparison of results in the literature difficult. The value of complication rates is limited unless the deformities are classified. To make comparison of treatment outcomes easier, Dahl et al. (1994) introduced a classification of the severity of the deformity.

Evaluation of our patient group with logistic regression analysis in a PLUM logistic regression model revealed that neither the severity classification nor the type of the deformity (malunion or (infected) nonunion) was related to the risk of complications. The percentage or proportionate bone loss of the affected bone was the only significant statistical factor that we could identify for the risk of complications. Resultant graphic representations of such analyses have can been constructed by other investigators, which makes comparison between authors possible.

When we break down the complications, the (re)fracture rate was 5 in 56 treated bone segments (9%), which is similar to the results of O'Carrigan et al. (2005) in a report on 986 lengthenings (8%). Also, all other local types of complications in our series were similar to those in most of the relevant literature (Antoci et al. 2006).

Psychological analysis and social support play an important role in patient selection and treatment (Tucker et al. 1990, Dendrinos et al. 1995, Garcia-Cimbrelo and Marti-Gonzalez 2004). Patients who are well prepared for the treatment and who do not have major psychological problems have better results (Ramaker et al. 2000). The mental and physical discomfort usually resolve after treatment (Hrutkay and Eilert 1990, Ghoneem et al. 1996, Ramaker et al. 2000). Mental condition is important in sustenance of the treatment, which was illustrated by the failure or severe complications in 2 patients and adapted treatment goals in 5 patients in our series.

Besides the type of deformity, the complication rate of the Ilizarov treatment is notoriously influenced by many other factors. It is known that surgeon's experience of more than 30 operations is needed to overcome the learning curve problems (Dahl et al 1994). This was not an issue in this study. Smoking was not evaluated as a determinant, because we had no information.

In conclusion, our study shows that the Ilizarov method is a valuable tool in treating severe types of bone loss and limb deformity with or without active infections. Reconstructive surgery using the Ilizarov method should always be considered as a treatment modality when amputation is imminent, though it is still difficult to judge when this type of reconstructive surgery is indicated (Bosse et al. 2002). Our analysis shows that the relative amount of bone loss from the affected bone or the relative amount of bone to be reconstructed dictates the complication rate. This is represented in a graph, which may be helpful for comparisons with published material involving similar reconstruction procedures.
